# Non‐Responders to Biologic Disease Modifying Antirheumatic Treatment for Inflammatory Bowel Diseases

**DOI:** 10.1155/mi/6977602

**Published:** 2025-12-10

**Authors:** Svetla Gadzhanova, Elizabeth Roughead

**Affiliations:** ^1^ Quality Use of Medicines and Pharmacy Research Centre, Clinical and Health Sciences, University of South Australia, Playford Building, Frome Road, Adelaide, South Australia, 5001, Australia, unisa.edu.au

**Keywords:** biologic DMARD, inflammatory bowel diseases, non-responders

## Abstract

**Background and Aim:**

Biologic disease‐modifying antirheumatic drugs (bDMARDs) are effectively used to relieve symptoms in inflammatory bowel diseases (IBD). The study aimed to examine the rate of non‐responders to bDMARD treatment in Australian population.

**Method:**

Cohort studies using a 10% random sample of the population dispensing medicines under the Australian Pharmaceutical Benefits Scheme (PBS). People aged 18 years and over who initiated bDMARD for IBD in 2019 or 2020 were followed for 12 months. The proportion of non‐responders (people who discontinued initial therapy by Week 16 and 40) was determined using Kaplan–Meier survival analysis.

**Results:**

There were 522 initiators of bDMARD for Crohn’s disease (mean age of 42 years). By Week 16, 15% discontinued initial therapy (primary non‐responders); 22% of initial responders discontinued bDMARD by Week 40 (secondary non‐responders). The primary non‐responder rate was lowest amongst infliximab initiators (6%), and highest for ustekinumab (24%). Infliximab had the lowest (17%) secondary non‐responder rate compared to the other biologics, suggesting less loss of response over time.

There were 390 initiators of bDMARD for ulcerative colitis (UC) (mean age of 44 years). By Week 16, 25% discontinued initial therapy; 21% of people with initial response discontinued by Week 40. The non‐responder rates were lowest amongst vedolizumab initiators (5% for primary and 8% for secondary) and highest amongst adalimumab (50% for primary and 48% for secondary).

**Conclusion:**

Comparison between bDMARD agents showed lowest initial non‐response and lowest loss of sustained response in infliximab initiators with Crohn’s disease and in vedolizumab initiators with UC.

## 1. Introduction

Crohn’s disease and ulcerative colitis(UC) are chronic inflammatory conditions that affect the digestive tract [1, 2]. The therapeutic goal in people with inflammatory bowel diseases (IBD) is to relieve symptoms and to induce and maintain clinical remission [3].

There are several biologic disease‐modifying antirheumatic drug (bDMARD) agents available in Australia for IBD. Tumour necrosis factor‐alpha (TNFa) antagonists are recombinant human immunoglobulin monoclonal antibodies that bind to TNF and inhibit its inflammatory action; they have been effectively used for IBD [4, 5]. In Australia, adalimumab and infliximab may be considered in moderate‐to‐severe Crohn’s disease and UC which is unresponsive to conventional treatment (for example with corticosteroids or 5‐aminosalicylates) [6, 7]. For Crohn’s disease, adalimumab and infliximab have been listed on the Pharmaceutical Benefits Scheme (PBS) since 2007–2008; for UC, adalimumab was listed on the PBS in 2016, while infliximab was listed on the PBS in 2014. Golimumab may be used for moderate‐to‐severe UC in adults who had inadequate response to conventional therapy [8] and was listed on the PBS in 2017.

For people who are intolerant to conventional therapies or TNFa antagonists, alternative therapies are available [9]. Vedolizumab is an integrin receptor antagonist for treatment of adult patients with moderate‐ to‐severe UC or Crohn’s disease who have had an inadequate response with, lost response to, or are intolerant to either conventional therapy or a TNFa antagonist [10]. It was listed on the PBS for both conditions in 2015. Ustekinumab is a monoclonal antibody that binds to and inhibits the biological activity of proinflammatory cytokines and interleukins involved in the pathophysiology of Crohn’s disease [11]. It was listed on the PBS for severe Crohn’s disease in late 2017.

Patients may receive an initial authority approval for 12 up to 16 weeks for biologic DMARD treatment for IBD [6–11]. The patient’s response to the initial course of treatment must be assessed and if there is a demonstrated response, then the therapy may be continued every 2 weeks for adalimumab [6], every 4 weeks for golimumab [8] and every 8 weeks for infliximab, vedolizumab and ustekinumab for up to 24 weeks under first continuing authority approval [7, 9]. Following a second assessment with demonstrated sustained response, the treatment may be continued as maintenance therapy for 24 weeks at a time under subsequent continuing authority approvals [6, 7, 9].

### 1.1. Aim of the Study

The aim of this study was to examine the rate of non‐responders to bDMARD treatment for IBD in Australian population.

## 2. Method

### 2.1. Data Source and Setting

De‐identified patient level data from a 10% random sample of the population from the Australian PBS prescription database of the Australian Government, Services Australia were utilised to identify prescriptions supplied for bDMARD agents between 1 January 2016 and 31 December 2021. Since mid‐2012, PBS data represent full capture of dispensing records for both general and concessional beneficiaries.

### 2.2. Study Design

Retrospective cohort studies were undertaken to investigate the rate of adult non‐responders to treatment with bDMARD agents for IBD, namely Crohn’s disease and UC.

### 2.3. Cohort Selection

The Crohn’s cohort was defined as people who were aged 18 years and over at the time when they initiated (first ever since 2016) a bDMARD medicine subsidised for Crohn’s disease between 1 January 2019 and 31 December 2020. The time period was chosen to allow for market uptake of golimumab and ustekinumab. Subjects were followed for 12 months postindex bDMARD. Similarly, the UC cohort was defined as people aged 18 years and over who received first ever (since 2016) bDMARD medicine subsidised for UC between 2019 and 2020; they were also followed for 12 months.

### 2.4. Exposure: Medicines Included in the Analysis

Medicines were coded in accordance with the World Health Organization’s Anatomical Therapeutic Chemical (ATC) classification system [12].

Biologic disease modifying antirheumatic agents for inflammatory bowel conditions included in the analysis: infliximab (L04AB02), adalimumab (L04AB04), golimumab (L04Ab06), ustekinumab (L04AC05) and vedolizumab (L04AA33).

### 2.5. Outcome Definition

Non‐responders were defined as:

a. Primary non‐responders—the proportion of all bDMARD initiators who discontinued initial treatment within 16 weeks to reflect lack of response to initial therapy and
b. Secondary non‐responders (loss of response over time)—the proportion of initial responders (people who continued index bDMARD at Week 16) who discontinued therapy within the next 24 weeks (by Week 40) to reflect lack of sustained response to therapy;

Discontinuation of initial (index) treatment episode was defined as either:

– Switch to a therapy not including the index medicine.
– Cessation—a gap in prescription refill for the index medicine based on the length of the estimated prescription duration which was calculated from the data and reflected the time period within which 75% of people returned for a repeat prescription for that medicine; gaps between index prescription and next refill which were more than three times the length of the estimated prescription duration were considered to represent discontinuation.

People who ceased index treatment but then reinitiated the same bDMARD therapy at a later time within the follow‐up period (i.e., had a break in therapy) were considered not to represent non‐responders and so were excluded from the analysis.

### 2.6. Statistical Analysis

Kaplan–Meier survival analysis was conducted to estimate the duration of the initial (index) treatment episode with bDMARD until discontinuation. People were followed up until discontinuation, death or end of follow‐up (1‐year postindex prescription). Persons who discontinued (switched or ceased) therapy before the end of follow‐up were reported as “event” persons. People who died before the end of follow‐up or continued therapy at 12 months were reported as “censored” people. Results were stratified by the type of the index bDMARD medicine.

Proportions were compared using chi‐square tests.

Analyses were performed using SAS 9.4 statistical package (SAS Institute, Cary NC, USA).

## 3. Results

### 3.1. Crohn’s Disease

**Figure 1:**
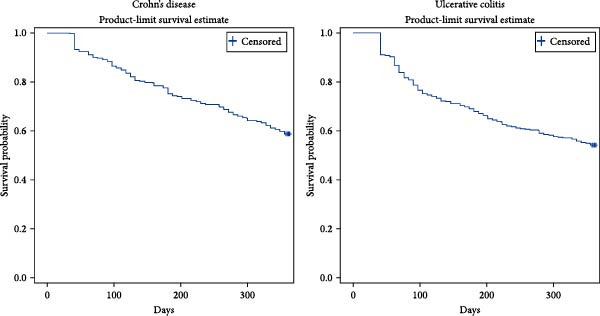
Kaplan–Meier estimate for time to treatment discontinuation of the first episode with index bDMARD.

**Figure 2:**
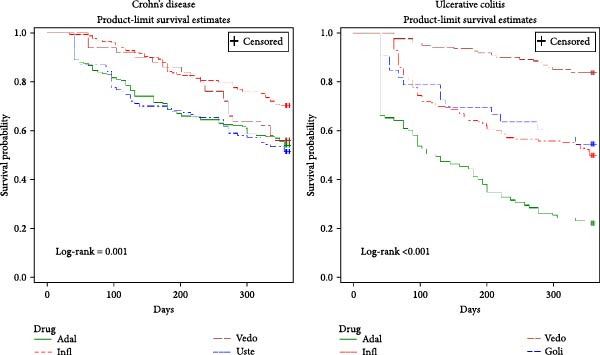
Kaplan–Meier estimate for time to treatment discontinuation of the first episode with index bDMARD—by index medicine. Abbreviations: Adal, Adalimumab; Goli, Golimumab; Infl, Infliximab; Uste, Ustekinumab; Vedo, Vedolizumab.

There were 544 people who initiated biologic DMARD for Crohn’s disease between 2019 and 2020, 4% of them had a break in therapy and were excluded, bringing the Crohn’s cohort to 522 people. Of these 522 people, 13% were dispensed bDMARD for fistulising Crohn’s disease. Most of the people in the cohort were initiated on adalimumab (38%), followed by infliximab (32%) and ustekinumab (20%). The mean age of the Crohn’s cohort was 42 years and more than half were females (54%) (Table 1). The median duration of initial therapy with any biologic DMARD was over 1 year (Figures 1 and 2).

**Table 1:** Demographic characteristics of bDMARD initiators (2019–2020).

Type of drug	Crohn’s disease			Ulcerative colitis		
	Number of people (%)	Mean age (SD)	Females	Number of people (%)	Mean age (SD)	Females
Any bDMARD	522 (100%)	42 (17.1)	54%	390 (100%)	44 (16.9)	52%
Adalimumab	200 (38%)	42 (17.1)	53%	95 (24%)	47 (17.1)	53%
Infliximab	165 (32%)	42 (17.1)	53%	140 (36%)	38 (15.7)	56%
Golimumab	N/A	N/A	N/A	33 (9%)	43 (15.9)	33%
Vedolizumab	50 (10%)	55 (18.8)	60%	122 (31%)	47 (17.0)	53%
Ustekinumab	107 (20%)	51 (15.5)	53%	N/A	N/A	N/A

Abbreviation: N/A, not applicable.

By Week 16, 15% of people in the Crohn’s cohort (*N* = 79) discontinued initial therapy with any bDMARD (Table 2). A breakdown of those primary non‐responders showed that 91% ceased and did not switch or reinitiate the therapy during the follow‐up period and 9% switched to a therapy not including the index medicine. When considering the type of index biologic agent for Crohn’s disease, three times more people who were initiated on adalimumab (20%) or ustekinumab (24%) did not respond to index therapy and discontinued by Week 16 compared to people who were initiated on infliximab (6%) or vedolizumab (8%). Chi‐square tests showed that primary non‐responder rates differed significantly between any two biologic agents (*p*  < 0.05) apart from infliximab and vedolizumab (6% and 8% respectively, chi‐square *p* = 0.649) and adalimumab and ustekinumab (20% and 24% respectively, chi‐square *p* = 0.432).

**Table 2:** Median duration of initial therapy and rates of primary non‐responders (discontinuation of therapy by Week 16) and secondary non‐responders (primary responders who discontinued therapy by Week 40).

Type of drug	Crohn’s disease			Ulcerative colitis		
	Median duration (95% CI)	By Week 16	By Week 40	Median duration (95% CI)	By Week 16	By Week 40
Any bDMARD	Over 1 year	15%	22%	Over 1 year	25%	21%
Adalimumab	Over 1 year	20%	23%	125 days (83; 188)	50%	48%
Infliximab	Over 1 year	6%	17%	Over 1 year	28%	23%
Golimumab	N/A	N/A	N/A	Over 1 year	21%	23%
Vedolizumab	Over 1 year	8%	30%	Over 1 year	5%	8%
Ustekinumab	Over 1 year	24%	22%	N/A	N/A	N/A

By Week 40, one‐fifth (22%) of people with initial response discontinued therapy with any bDMRAD (secondary non‐responders), with the lowest secondary non‐responder rates amongst infliximab initiators (17%) and the highest amongst vedolizumab initiators (30%) (Table 2). The secondary non‐responder rates between any two biologic agents did not differ significantly.

### 3.2. Ulcerative Colitis

There were 440 people who received an index bDMARD for UC in the period 2019–2020. The UC cohort consisted of 390 people after excluding people who had a break in therapy. In the UC cohort, the most used medicine at initiation was infliximab (36%), followed by vedolizumab (31%) and adalimumab (24%). The mean age of all people in the cohort was 44 years and 52% were females (Table 1). The median duration of initial therapy was over 1 year except for index adalimumab (Figures 1 and 2).

By Week 16, 25% of people with UC (*N* = 99) discontinued initial therapy with any bDMARD (Table 2). From these primary non‐responders, 86% ceased and did not switch or reinitiate the therapy during the follow‐up period and 14% switched to a therapy not including the index medicine. When stratified by index medicine for UC, only 5% of people who were initiated on vedolizumab discontinued index therapy by Week 16 compared to over one fifth of people who were initiated on golimumab (21%) and infliximab (28%) and half of those initiated on adalimumab (50%). Chi‐square tests showed that primary non‐responder rates differed significantly between any two biologic agents (chi‐square *p*  < 0.005) apart from infliximab and golimumab (28% and 21% respectively, chi‐square *p* = 0.547).

By Week 40, one‐fifth (21%) of people with initial response discontinued therapy with any biologic DMARD, with the lowest secondary non‐responder rates amongst vedolizumab initiators (8%) and the highest rates amongst adalimumab initiators (48%) (Table 2). The secondary non‐responder rates between any two biologic agents differed significantly (chi‐square *p*  < 0.05) apart from infliximab and golimumab (23% and 23% respectively, chi‐square *p* = 0.979).

## 4. Discussion

For Crohn’s disease, this study found that the proportion of primary non‐responders to any biologic DMARD (people who discontinued initial therapy by Week 16) was 15%, as no authority approval was received to continue initial bDMARD beyond Week 16. From people with initial response (on therapy at Week 16), one‐fifth discontinued treatment with any biologic DMARD by Week 40. The lack of demonstrated sustained response in the secondary non‐responders means that no subsequent authority approval was received to continue therapy as maintenance therapy beyond Week 40. The reason for cessation or failure to trial an alternative therapy is unknown as this is not recorded in the dataset, but could related to side effects or patient experience. Patients may also have reinitiated a therapy subsequent to our follow‐up time.

When considering the type of the index biologic agent for Crohn’s disease, primary non‐responder rate in infliximab and golimumab initiators was 6% and 8% respectively, which is three times lower than the rates for adalimumab and ustekinumab (20% and 24% respectively). Our results are unadjusted for patient characteristics but are similar to a cohort study by Schnitzler [13] reporting 11% of primary non‐response to initial therapy with infliximab within 10 weeks and another cohort study by Swoger [14] reporting 18% primary non‐response rate to initial adalimumab. A recent systematic review and network meta‐analysis has looked at 15 randomised controlled trials (RCTs) in adults naïve to biologic therapies for moderate to severe Crohn’s disease [15]. The authors compared the efficacy of several biologic agents in terms of induction of clinical response after duration of therapy of minimum 2 weeks. They found that infliximab was associated with significantly higher odds of inducing clinical response compared to adalimumab (OR = 8.84, CI 1.95–40.03) and ustekinumab (OR = 7.90, 95% CI 1.78–35.10) which is in accord with our findings. However, they did not find vedolizumab to be associated with significantly higher odds compared to adalimumab and ustekinumab [15].

We found that even though infliximab had lower secondary non‐responder rate (17% compared to 22%–30% for the other biologics) it was not statistically significant. The systematic review and meta‐analysis by Singh [15] also found that even though infliximab had slightly better efficacy for maintenance of clinical remission compared to ustekinumab and vedolizumab the odds ratio results were non‐significant. The authors concluded that no biologic agent was superior to others as maintenance therapy when the duration of therapy was for at least 22 weeks. Infliximab was only available in the infusion form during the study period, with subcutaneous formulations first listed on the Australian PBS in July 2021. Our findings on 1‐year persistence stratified by the type of biologics for Crohn’s disease are similar to another Australian study which found persistence rates over 60% at 1 year for ustekinumab, vedolizumab, infliximab and adalimumab [16].

For UC, our study found that the proportion of primary non‐responders to any bDMARD (people who discontinued initial therapy by Week 16) was 25%. One‐fifth of people with initial response (on therapy at Week 16) discontinued bDMARD by Week 40.

Stratification by the type of the index biologic agent for UC showed that the primary non‐responder rate in vedolizumab initiators was 5%. It was significantly lower compared to over one‐quarter of infliximab (28%), one‐fifth of golimumab (21%) and half of adalimumab initiators (50%). Our results for infliximab and adalimumab are similar to two RCTs reporting 31% primary non‐response for infliximab [17] and 50% primary non‐response for adalimumab [18] at Week 8. A meta‐analysis of 15 RCTs on biologic‐naïve patients with moderate‐severe UC found that the effect size for induction of clinical remission was strongest for infliximab (OR 4.07, 95% CI 2.68–6.16) and vedolizumab (OR 3.10, 95% CI 1.53–6.26] compared to placebo [19]. However, there were no significant differences between the efficacy of different biologic agents on clinical remission after initial treatment [19].

Secondary non‐responder rates were lowest amongst vedolizumab initiators (8%) and highest amongst adalimumab initiators (48%). This suggests that vedolizumab was associated with less loss of response over time. A Phase 3 double‐blind trial found vedolizumab (vs. placebo) to be effective maintenance treatment in people with moderate to severe UC, with a good safety profile [20]. Vedolizumab was also found to be superior to adalimumab at achieving clinical remission at Week 52 in another RCT [21]. A recent meta‐analysis reported vedolizumab to have the highest persistence at 1 and 2 years in UC [22].

Our 1‐year persistence stratified by the type of biologics for UC showed highest rates for vedolizumab and lowest for adalimumab similar to another Australian study which also found highest persistence at 1 year for vedolizumab (73%) and lowest for adalimumab (46%) [16].

During the study period, prescribing of originator bDMARDs in Australia a written authority approval was required for both initial and continuing prescriptions, with prescribers required to document auditable criteria at the time of the initial and continuing authorities to provide evidence that patients were responders to therapy. Biosimilar products required written authorities for initial prescribing and the first continuing prescription, but subsequent continuation prescriptions were able to be prescribed under a streamlined authority, which does not require the proof of response. Biosimilars for infliximab were available from 2017 onwards, while adalimumab biosimilars were introduced in March 2021. It is possible that the authority prescription process played some influence on the results observed.

There are several limitations of the study. Even though the data include all dispensed medicines, we are not able to ascertain if the medicines were actually taken. Concomitant use of other immunosuppressive medicines was not investigated and may have influenced the observed results. The data does not provide information on disease severity or any measures for clinical remission. Our analyses were not adjusted for patient characteristics, which may have influenced decisions on choice of therapy. Further, we had no information on cessation due to adverse events or switching due to patient or doctor preference for administration mode (e.g., infusion over subcutaneous administration) or doctor preferred therapy.

Reinitiation of therapy is possible under the PBS listings, with criteria for reinitiation varying on whether there has been a treatment break in biological therapy of more or less than 5 years. Infliximab and adalumimab have been available in Australia for a number of years prior to our study period and its possible some initiators in our study had these therapies prior to the study start date. Vedolizumab entered the market in 2015, ustekinumab in 2017 and golimumab in 2018, some of this use may have been reinitiation and that may partly explain the older average age observed for vedolizumab and ustekinumab in the Crohn’s population.

## 5. Conclusion

Our results show high continuation rates at 1 year with initial bDMARDs for Crohn’s and UC. Comparison between biologic DMARD agents showed that using discontinuation as a proxy measure of non‐response, lowest initial non‐response and lowest loss of sustained response was in infliximab initiators with Crohn’s disease and in vedolizumab initiators with UC.

## Ethics Statement

The study used de‐identified data and conforms to management and release of data in accordance with the principals of the Australian Government Privacy Act, 1988. It has an ethics approval by the External Request Evaluation Committee (RMS3527). The study uses personally non‐identifiable data and thus we did not obtain informed consent for participation.

## Disclosure

All authors read and approved the final manuscript.

## Conflicts of Interest

The authors declare no conflicts of interest.

## Author Contributions

Both authors contributed to the study conception and design. Data analysis were performed by Svetla Gadzhanova. The first draft of the manuscript was written by Svetla Gadzhanova and both authors commented on previous versions of the manuscript.

## Funding

This study was funded under the Australian Government Department of Health Value in Prescribing Initiative.

## Data Availability

PBS data is available under licence at the request of the data custodian.
